# Culture Matters in Communicating the Global Response to COVID-19

**DOI:** 10.5888/pcd17.200245

**Published:** 2020-07-09

**Authors:** C.O. Airhihenbuwa, J. Iwelunmor, D. Munodawafa, C.L. Ford, T. Oni, C. Agyemang, C. Mota, O.B. Ikuomola, L. Simbayi, M.P. Fallah, Z. Qian, B. Makinwa, C. Niang, I. Okosun

**Affiliations:** 1 School of Public Health, Georgia State University, Atlanta, Georgia; 2 College for Public Health and Social Justice, Saint Louis University, Saint Louis, Missouri; 3 Department of Community Medicine, Midlands State University, Gweru, Zimbabwe; 4 Center for the Study of Racism, Social Justice & Health, UCLA Fielding School of Public Health, Los Angeles, California; 5 MRC Epidemiology Unit, University of Cambridge, Cambridge, United Kingdom, and School of Public Health and Family Medicine, University of Cape Town, South Africa; 6 Department of Public Health, Amsterdam UMC, University of Amsterdam, Amsterdam Public Health Research Institute, Amsterdam, Netherlands; 7 Department of Public Health, Institute of Collective Health, Federal University of Bahia, Salvador, Brazil; 8 Human Sciences Research Council, Cape Town, South Africa; 9 National Public Health Institute of Liberia, Office of the Director–Monrovia, Greater Montrovia, Liberia; 10 AUNIQUEI, Office of the Director and Chief Executive Officer–Lagos, Lagos, Nigeria; Former Director of African Region of United Nations Population Fund; 11 Institute of Environmental Sciences, Cheikh Anta Diop University, Dakar, Senegal

## Abstract

Current communication messages in the COVID-19 pandemic tend to focus more on individual risks than community risks resulting from existing inequities. Culture is central to an effective community-engaged public health communication to reduce collective risks. In this commentary, we discuss the importance of culture in unpacking messages that may be the same globally (physical/social distancing) yet different across cultures and communities (individualist versus collectivist). Structural inequity continues to fuel the disproportionate impact of COVID-19 on black and brown communities nationally and globally. PEN-3 offers a cultural framework for a community-engaged global communication response to COVID-19.

**Summary**

**What is already known on this topic?**

The World Health Organization developed risk communication and community engagement (RCCE) to facilitate global response to COVID-19. RCCE communicates about individual risks but communicates little about community risks.

**What is added by this report?**

Community engagement requires knowledge of culture in framing COVID-19 communication and messaging. The PEN-3 cultural model was used to frame community engagement for collective actions.

**What are the implications for public health practice?**

COVID-19 reveals existing structural inequity in black and brown communities nationally and globally. PEN-3 offers a cultural framework for community-engaged communication and messaging for COVID-19.

## Introduction

Our primary aim in this commentary is to offer a community-engaged communication strategy that focuses on coronavirus disease 2019 (COVID-19) messages in cultural context. COVID-19, the disease caused by the novel severe acute respiratory syndrome coronavirus 2 (SARS-CoV-2), was declared a global pandemic on March 11, 2020. Since that time, messages of prevention have focused primarily on preventing individual risks, particularly for those with preexisting chronic conditions, including hypertension, diabetes, stroke, and asthma. As infection and death rates grow, communication about response to the pandemic has increasingly focused on individual behavior choices, which assumes that prevention is largely in an individual’s control. In efforts to promote uniform messaging for COVID-19, the World Health Organization developed a multilevel risk communication and community engagement (RCCE) response strategy for health care workers, the wider public, and national governments (1,2). 

Well intentioned as RCCE may be, the strategy ends up focusing more on individual risk and less on community engagement. By community engagement, we mean creating spaces and opportunities for those who live in the community to have their voices heard in naming the problem and offering solutions to the problems they face (3). The process of such engagement also includes identifying community resilience and ways to build on values that are important to the community. Communication about individual risk is important, but prevention and control messaging is more likely to be achieved when we engage the voices of those who live in the communities, particularly communities that bear the heaviest burden of the pandemic. 

Vulnerability to the COVID-19 pandemic cannot be fully explained by individual risks alone but rather by broader social and structural determinants of health that result in inequities in communities where vulnerable populations live, work, play, pray, and learn (4–6). Moreover, a disproportionate burden of COVID-19 mortality is among racial and ethnic populations in communities that have had historical inequities in health (7–9). With increasing global mortality, a deep concern remains about the alarming levels of general spread, disease severity, and inaction for these communities (10). Research on health disparities, particularly on antiracism (11), demands a focus on risk environment and risk situation rather than the conventional epidemiologic focus on risk factor, which tends to place the burden of behavior change on individuals rather than the context and structure that define and confine their vulnerability (12–14). Thus, community-engaged communication is crucial for acknowledging the voices of those in the community with culturally relevant solutions that are more likely to be sustained beyond the pandemic. Communities that are the most affected experience historical, structural inequities that create not only their preexisting chronic health conditions but also their preexisting vulnerable living and working conditions (15). To understand these communities, the role of culture matters if any communication strategy is to be adopted or sustained.

## Culture and Communication for Health

Culture is central to effective COVID-19 messaging for community engagement. We define culture as a collective sense of consciousness that influences and conditions perception, behaviors, and power and how these are shared and communicated (3). Culture may appear neutral, but its power to define identity and communities as a collective is based on values expressed through institutions such as health care, education, and families (3). Culture shapes language, which in turn shapes communication both in message delivery and reception. In response to COVID-19 in Europe, for example, cultural sensitivity to racial and ethnic minority group experiences is believed to be critical if messages for mitigation are to have broader impact (16). 

Framing communication messaging that engages the most affected communities can draw some lessons from the multilevel strategies employed in HIV communication, which identify relevant structural factors of institutional policy, economic status, gender, and spirituality while grounded in the force of culture (17,18). For example, as part of HIV communication strategy, the concept of “zero grazing” was introduced in Uganda as a prevention message for multipartner marriages by encouraging that sexual activities be kept within the circle of those in the marriage only. This message was a community collective response to the conventional individualist message of one-to-one sexual relations. 

For COVID-19, some black and brown communities have initiated collective communication for mitigation so that messages have cultural meanings for those with whom they share common cultural values. For example, although heavily affected by COVID-19, some indigenous communities in the United States have sought their own solutions to this pandemic by using traditional knowledge and language to promote voluntary isolation at the individual level and sealing off their territories at the community level (19) while still being able to continue aspects of their spiritual well-being (20). Thus, to rapidly improve our communication messages in response to COVID-19, we need an effective global response that invites community-engaged solutions with culture as a connecting space.

Culture is key to the global response to community engagement. COVID-19 unveils a pattern of cultural insensitivity that has also been evident in communication about Ebola. In the early stages of the Ebola outbreak in 2014–2015, conventional messages did more harm than good because they did not value the cultural roles associated with death. Two examples of these messages were, “When you get Ebola, you will die” or “If someone is sick, don’t touch him.” In Liberia, the high death rate from malaria and other diseases among the poor blunted messages for urgency to heed prevention and treatment of Ebola (21). In the West Point slum of Monrovia, Liberia, for example, adhering to physical distancing for Ebola and now COVID-19 is made difficult by sea erosion from the past 10 years, which reduced the land mass by 50%, even though the same number of people remain. Structural inequities often reveal the limit of individual choices in the absence of corrective actions to address contextual constraints over which the community has no control. These constraints are the preexisting contexts of inequities in many black and brown communities globally (5,22). 

We believe that COVID-19 mitigation efforts that focus on individual behavior such as handwashing and physical distancing must be balanced with structural mitigation efforts such as clean water, access to housing, unemployment, and for those with jobs, ability (type of job) and tools (access to computer and internet) to work from home. These are the daily realities of racial/ethnic and economically disadvantaged populations that bear the heaviest burden of the pandemic (22). Yet as has been learned from HIV (23) and Ebola (21), culture offers communication messaging that ranges from positive aspects of lived experience that should be promoted to negative practices that should be overcome within the context of communities. To frame approaches to communications and community engagement for COVID-19, we use the PEN-3 cultural model (Figure). We believe that this model offers a roadmap for engaging communities in communication about COVID-19 mitigation efforts.

**Figure:**
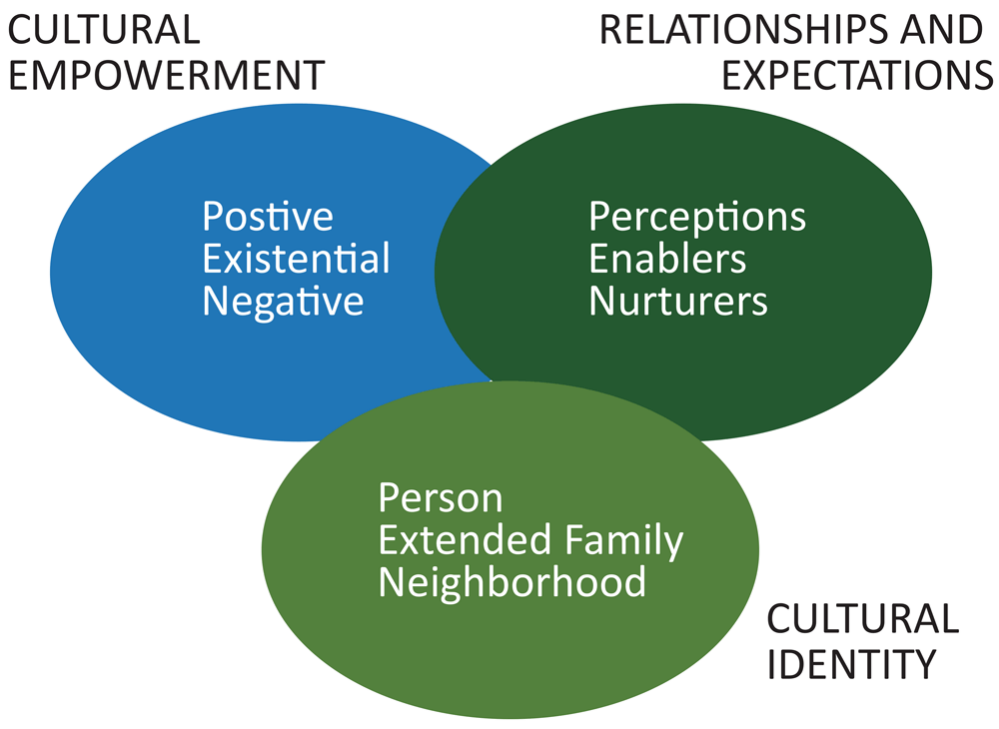
The PEN-3 Model. The model has 3 primary components: cultural identity, cultural empowerment, and relationships and expectations, and each of the 3 components has 3 domains.

## PEN-3 Model and Communication Response to COVID-19

PEN-3 is a cultural model that was developed and first published in 1989 (24). The PEN-3 cultural model consists of 3 primary domains: 1) cultural identity, 2) relationships and expectations, and 3) cultural empowerment. Each domain includes 3 factors that form the acronym PEN; person, extended family, neighborhood (cultural identity domain); perceptions, enablers, and nurturers (relationship and expectation domain); positive, existential and negative (cultural empowerment domain). The domains are described in detail elsewhere (3). A key outcome of using PEN-3 is learning to first identity the positive aspects of behavior and culture such that negative behavior is not the only focus of intervention, as shown in a systematic review (25). At the height of the global HIV stigma and racism against the cultures of black and brown identities, PEN-3 was developed to offer a space for voices to be heard that are otherwise silenced. The model was designed to guide researchers and practitioners to listen to those voices, and in so doing, to ask for not only what these communities were doing wrong but to begin with what they are doing correctly. Culture exists where we live, work, play, pray, and learn. In PEN-3, the focus on cultural logic of decision making about a pandemic is less about who is right or wrong than about what societal reasoning and rationale are at the foundation of the message. Even more important is which populations and communities are the intended audience for messages meant to be solutions. Thus, the importance of the positive aspects of a community and people, their collective resilience, and their cultural logic must not be overshadowed by the presence of diseases, as we have learned from the work on HIV and Ebola and now COVID-19. Therefore, reframing COVID-19 communication messages globally must respond not only to individuals but to the community as a collective. Individuals must not be privileged over the collective or community.

Science also has culture. The application of the PEN-3 model to COVID-19 communication also applies to the scientific community whose task it is to solve the disparities unveiled by COVID-19. To acknowledge that the scientific community exists within 1 or more cultures is to remove it from the pedestal on which it has rested for so long in ways that are well beyond any reproach and critique of the notion that science is inherently value-free (26). Indeed, questions about the effectiveness of social distancing have contrasting beliefs between a country like Sweden (which does not believe in social distancing) and the United States (which does); yet both are based on scientific claims, confirming that science is itself a production of culture and politics. In focusing on the PEN-3 domain of cultural empowerment, for example, the positive and existential dimensions of scientific culture are eagerly and frequently promoted by the scientific community. However, the negative dimensions evident in contrasting recommendations must also be examined, because they create communication challenges. To remedy the challenges requires messaging that promotes cultural inclusivity in the responses to the COVID-19 pandemic. 

For years, science ignored the role of structural racism in explaining and predicting disease burdens. Yet it is structural racism that created and maintains communities in which preexisting chronic health conditions such as hypertension and diabetes exist. Therefore our communication should address actions we take at the individual level, risks we face at the collective and community level, and the role science plays in promoting or hindering mitigation efforts. Thus, for COVID-19, PEN-3 offers the importance of cultural empowerment anchored in community-engaged mitigation efforts. We need to focus on both individual risks and community engagement and in so doing address 3 binarisms that must be coalesced to advance global communication for COVID-19. To illuminate the power of culture in community engagement, each of the PEN-3 domains is paired with a binary that needs to be understood and coupled in communication about COVID-19.

## Preexisting Chronic Conditions and Preexisting Structural Contexts: Cultural Empowerment

Whereas the language of risk factors focuses on individual preexisting chronic conditions such as diabetes, hypertension, and asthma, the language of health disparities and risk environments focuses on preexisting community contexts. These include unhealthy food structures, unemployment environments, poor housing (eg, intergenerational cohabitation), and job types that define and confine vulnerability to COVID-19. The language of individual risk has been used to frame the prevention message of social distancing and wearing a mask. Yet, a recent commentary concluded that physical distancing is a privilege for populations with preexisting contexts that reinforce not only vulnerability to conditions like diabetes but also living conditions that make it impossible to adhere to physical distancing (27). Several recent publications have emerged in which scholars have lamented the heavy racial burden of COVID-19 on African American, Latino, and Native American populations in the United States (8,9,28). Similar alarm has been raised in Europe, particularly among immigrant populations (16) and in Brazil, which has one of the highest number of cases in the world. In Brazil, nearly 6% of the population, which is mostly black, live in favelas (slums or shantytowns located within or on the outskirts of the country’s large cities) and are exposed to social and environmental vulnerability with poor access to water and employment, among other needs (29). Socio-spatial inequality determines the patterns of Brazilian cities and the disposition of housing conditions, which limit adherence to the health policy of social isolation. This accumulation of disadvantages represents structural risks for any health condition, which has resulted in high prevalence of many neglected diseases in these vulnerable areas in Brazil. In South Africa, particularly in the absence of official data based on race/ethnicity, the government downplayed racial/ethnic vulnerability until the premier of the Province of Gauteng, which includes Johannesburg, revealed that the hotspots of COVID-19 in his province were shifting from the suburbs, where most whites live, to townships, where most blacks and people of mixed race (known as coloreds) live (30). In many Nigerian cultures, certain cosmological viewpoints suggest that fate determines diseases and ill health and that these are independent of science and human actions (31). The cultural empowerment domain of the PEN-3 model allows COVID-19 interventionists to look at the total context, including how people construct their lived experience within their resilience and the hurdles in their communities. COVID-19 communication should begin with positive factors, such as persistence and resilience, to achieve solutions that nurture and revive the community. To better understand the role of culture in a pandemic we can draw lessons from 2 pandemics that remain with us today, HIV and Ebola (Table).

**Table:** Application of the PEN-3 Cultural Model to COVID-19, Ebola, and HIV

PEN-3	COVID-19	Ebola	HIV
Perceptions	++Knowledge about 80% exposure with little or no illness ==Pandemic affected all countries, rich and poor –Awareness did not translate into action for prevention, therefore the need to modify messages	++Knowledge of virulence of the disease ==Pandemic affected mostly West and Central Africans –Awareness did not translate into behavior change, therefore messages had to be modified to fit cultural context	++Knowledge of behaviors that lead to vulnerability ==Different contexts and factors of vulnerabilities –Awareness did not translate into behavior change
Enablers	++Availability and use of protective personal equipment, such as masks and gloves ==Traditions like burial were partly affected –Health care providers do not have all the support they need to care for those infected	++Availability and use of protective personal equipment, such as masks and gloves ==Traditions like burial were fully and directly affected –Health care providers do not have all the support they need to care for those infected	++Availability of male and female condoms and needle exchange programs ==Traditions like marriages were directly affected –Health care providers do not have all the support they need to care for those infected
Nurturers	++Family members caring for loved ones even when there is risk ==Cultural identity–based messaging about community inequities as response to COVID-19 and noncommunicable diseases –Family members losing their jobs and not being able to provide basic needs for loved ones	++Family members caring for loved ones even when there is risk ==Culture-based solution such as traditional leaders (eg, chiefs overseeing burial rites) –Family members losing their jobs and not being able to provide basic needs for loved ones	++Family members caring for loved ones even when there is risk ==Culture-based messages such as monogamy for individualists and “zero grazing” for collectivist contexts –Job discrimination against those infected

Key: ++ positive to be promoted; == existential to be recognized; – negative to change.

## Individualist Versus Collectivist: Cultural Identity

Every society has a social contract that frames the ways we act and prioritize decisions and choices: as individuals, such as in the United States, as the collective as in China, or some mix of those forms as in Canada and France. One of the key lessons for a global response to a pandemic is that the cultural logic of different societies shapes and influences their prevention strategies. In the United States, individual vulnerability to risk is culturally privileged over community risk, when both should be addressed equally. Such coalescing of dual logics is embodied in the cultural messages from the yin and yang (coexistence and balancing of opposite forces) that may inform messaging in China; Ubuntu (I am because we are) in South Africa; and the expression “Nit nittay garabam” (The person is the remedy of the person) in Wolof in Senegal (32). These cultural expressions are different, neither better nor worse than individualist cultural logic that typically informs messaging in the United States. In China, for example, quarantine was implemented in Wuhan as a collective action to varying degrees and scopes. At the individual level, everyone was mandated to stay at home, and a permit to leave home could be obtained only from a community committee made up of volunteers. At the city level, all city entries and exits were screened; all public transport was discontinued including public bus, subway, ferry, and taxi. This response reflected the collectivist social and cultural contract of Chinese society (33). Thus, when a message of response in one country is communicated in another as draconian, for example, we need to unpack the different rather than competing cultural logics that inform these messages, particularly in a pandemic. Given the virulence of COVID-19, communication messages must be inclusive of multiple cultural logics whereby the word “and” is preferred over the word “or”. In the book entitled *Built to Last* (34), the authors debunked the competing binarism of and/or in their study of the characteristics of successful and enduring visionary companies. In advancing the phrases, the “tyranny of the or” and the “genius of the and,” the authors made the case for why duality is a strength and not a competition in which one side has to win. COVID-19 messaging globally should embrace cultures and communities with the genius of the “and” by not privileging any one culture over another. The late Chinua Achebe, a Nigerian novelist, once noted that for collective cultures, wherever one idea stands, it is absolutely necessary to expect another idea to stand next to it (35). Thus, instead of thinking in single cultural logic, we have to embrace multicentric logics – individual, collective, and everything in between.

## Noncommunicable Diseases and COVID-19: Relationship and Expectation

As the world is consumed with the COVID-19 pandemic, there remains a silent pandemic of noncommunicable diseases (NCDs) that now coexist in the same communities most affected by COVID-19. The response to NCDs in the context of COVID-19 should remain a top priority as part of structural solutions to inequities. To promote equity, we must address the structural determinants of health by first addressing structural racism, which is inscribed in institutional policies and practices that have created and sustain the disproportionate burden of hypertension, diabetes, and other NCDs in the black and brown communities (5). Thus, structural racism is a key determinant of such NCDs as hypertension, diabetes, stroke, and asthma (6). NCDs are the leading cause of death worldwide, with the most significant burden placed on low-income and middle-income populations in terms of premature deaths. In the United States, racial minorities, specifically black, Latino, and Native American populations, are the most burdened by NCDs (36). Indeed, the leading causes of death in these populations are heart disease, cancer, unintentional injuries, chronic lower respiratory disease, stroke, and cerebrovascular diseases, which together account for approximately 65% of total deaths (37). Thus, the NCD burden exists in the same population where COVID-19 exists. Our communication messaging, therefore, should erase a binarism of competition that leads to a pandemic *or* NCDs rather than COVID-19 *and* NCDs. The behaviors and context that favor one condition are likely to favor the others. Indeed, where NCD stands, infectious diseases like COVID-19 are likely to stand next to it. The messages of COVID-19 prevention in social and physical distancing and wearing masks are important solutions, but their sustainability depends on adequate response to disparities in the burden of diabetes, asthma, and other NCDs that are preexisting chronic conditions. Structurally, social distancing is problematic in South African townships, Brazilian favelas, and Nigerian slums where people share with one another basic essentials, such as sugar or salt when they run out of stock. The situation is further exacerbated by the lack of access to potable water in many of these communities including the *quartiers* of Senegal, the town of Khayelitsha in South Africa, favelas in Brazil, slums of Nigeria, and Flint, Michigan, in the United States. Communication and messaging for COVID-19 should also focus on us as health scientists and professionals by looking to ourselves for the same needed cultural transformation that we expect from communities responding to NCD pandemics as we do for infectious pandemics. Similar to Ebola (38) and HIV, COVID-19 revealed the falsehood in the separation of disease burdens by how they come to inhabit our bodies. This is the time for communication and messaging to focus not only outward to the community but also inward toward public health experts who frame the messages. How we respond now to COVID-19 is how we must respond to NCDs like hypertension, diabetes, obesity, cholesterol management, and asthma, because these disorders are constant reminders of persistent inequities in our communities.

## Implications for Public Health

COVID-19 communication and messaging should address community risks at least as much as individual risks. PEN-3 offers a communication framework that engages the community by promoting positive factors, acknowledging unique factors, and preventing negative factors. There is a limit to the culture(s) of science, and scientists should reexamine the negative dimensions of scientific cultural solutions to the pandemic. Research and evaluation are also needed to embrace alternative perspectives and the culture of policy and politics that influence the choice of architecture for communication and messaging strategies. Such research and evaluation, for example, on communicating risk mitigation, should democratize scientific research and empower communities to advance solutions to the root causes of health inequities and strategies to improve their own well-being (39). By offering a model for effectively engaging communities, PEN-3 also focuses on mutual community-centered strategies, highlighting not only the perceptions that matter but also the enablers or resources and nurturers or collective roles that foster community agency and voice in mitigating the COVID-19 pandemic. Moreover, to the extent these strategies center equity, they enable culturally grounded approaches to scientific inquiry and challenge the field from within itself to honor community agency and resilience. These alternative perspectives can accelerate efforts in health equity by identifying and addressing the underlying structural determinants of inequities, such as structural racism, that lead to the disproportionate burden of COVID-19 cases and deaths among racial/ethnic minority groups. Ultimately, the goal of COVID-19 communication and messaging within culture is to mitigate increase in new cases and deaths, address preexisting structural contexts, and ultimately advance global communication messaging that promotes health and social justice for this pandemic now and others in the future.
